# Amikacin bladder instillation as prophylaxis for recurrent urinary tract infections in a child with neurogenic bladder: A case report

**DOI:** 10.1016/j.idcr.2026.e02651

**Published:** 2026-06-22

**Authors:** Afnan Alsunaidi, Abdulaziz A. Alsalem, Yara A. Aldajani, Sarah Alsubaie

**Affiliations:** a Pharmacy Department, King Saud University Medical City, King Saud University, Riyadh, Saudi Arabia; b Pharmacy Department, Security Forces Hospital, Riyadh, Saudi Arabia; c Department of Pediatric, College of Medicine, King Saud University, Riyadh, Saudi Arabia

**Keywords:** Pediatrics, Urinary Tract Infection, Recurrent Urinary Tract Infection, Intravesical, Amikacin

## Abstract

**Background:**

Recurrent symptomatic urinary tract infections (rUTIs) in pediatric patients with neurogenic bladder and chronic kidney disease can be particularly difficult to manage with an appropriate prophylaxis and treatment, especially when caused by multidrug-resistant organisms. Intravesical aminoglycoside instillation may provide a safe renal-sparing prophylactic option.

**Case presentation:**

A 9-year-old female with neurogenic bladder due to voiding dysfunction, stage 2 chronic kidney disease and bilateral hydronephrosis, who presented with high-grade fever, flank pain and vomiting. She had had 3 hospitalizations for multi-drug resistant urinary tract infections (MDR-UTIs) in the past 6 months, despite previous use of oral prophylaxis. Initially, she was receiving sulfamethoxazole/trimethoprim, then switched to nitrofurantoin for 6 months. The urine culture grew Klebsiella pneumoniae (ESBL-producing), with sensitivity to amikacin, meropenem and imipenem. The patient was treated with intravenous meropenem for 14 days, with both clinical and microbiological resolution. To minimize the chance of recurrence, a prophylaxis regimen started as intravesical amikacin instillation (250 mg in 50 mL normal saline) via self-intermittent catheterization – daily for 2 weeks, alternate days for 10 weeks, and finally twice weekly for 12 weeks. At 2-week follow-up visit, she was additionally started on prophylactic sulfamethoxazole/trimethoprim. Approximately 18 months follow-up after discharge, the patient remained asymptomatic with no further UTIs.

**Conclusion:**

This case report provides useful information on effective long-term prophylaxis of multi-drug resistant recurrent urinary tract infection or rUTI, using intravesical amikacin in a child with neurogenic bladder and renal injury. Utilizing specific protocols for dosage standardization and prospective studies to assess the safety and effectiveness would be beneficial.

## Introduction

Multiple recurrent Urinary tract infections (UTIs) are common, with over one-third experiencing recurrence after their first episode, especially those with neurogenic bladder [1]. Preventing recurrent UTI in such children includes clean intermittent catheterization, antispasmodics and antibiotics [1]. In neonates, amoxicillin is the agent most frequently used for continuous antimicrobial prophylaxis, while in older children trimethoprim-sulfamethoxazole (TMP-SMX) and nitrofurantoin are most frequently used [2]. Neurogenic bladder (NB) exhibits several risk factors for UTI, including increased bladder pressures that predispose individuals to vesicoureteral reflux, bladder ischemia, and stasis [3]. Recurrent UTIs in affected children elevate the risk of urosepsis, which has a mortality rate of 10–15%, as well as the risk of end-stage renal disease (ESRD) [4]. The treatment is unnecessary for asymptomatic bacteriuria; however, symptomatic recurring UTIs caused by MDR organisms are challenging to manage. Options for antimicrobial treatment are constrained due to antibiotic resistance [5]. Consequently, intravesical antibiotic treatment may serve as an effective alternative for treating and/or preventing recurrent UTI due to elevated local antibiotic concentrations [6]. There is a limited literature to support the selection of intravesicular antibiotics in this population. This case report describes the use of intravesicular amikacin instillattion to prevent UTI in a pediatric patient with neurogenic bladder dysfunction.

## Case report

A 9-year-old female was admitted to the general pediatric ward with a history of fever, high-grade (39 °C) for one day, shivering, flank pain, and vomiting. The previous medical history was significant for neurogenic bladder secondary to voiding dysfunction, chronic kidney disease stage 2, and Bilateral hydronephrosis on clean intermittent catheter (CIC). She also underwent a Botox injection two days before admission. In the preceding 6 months, she had 3 episodes of UTIs and pyelonephritis with multidrug-resistant organisms requiring hospitalizations and intravenous antibiotics. For UTI prophylaxis, she was receiving sulfamethoxazole/trimethoprim (Bactrim DS) 80 mg orally once daily, then switched to nitrofurantoin 100 mg orally twice daily (2.5 mg/kg/day) for 6 months. Other medications were administered orally and included: lisinopril 5 mg daily, solifenacin 5 mg once daily, and sodium bicarbonate 325 mg (3.8 mmol) twice daily. The patient also received cholecalciferol 10,000 units orally every week.

The physical examination at admission was unremarkable. At the time of admission, she weighed 40 kg and had a serum creatinine level of 97 mcmol/L (1.1 mg/dL), with an estimated creatinine clearance of 51 mL/min. The patient was afebrile (36.5 °C), and vital signs normal. The basic metabolic panel was normal except for elevated blood urea nitrogen 10 mmol/L (28 mg/dL), serum creatinine 97 mcmol/L (1.1 mg/dL). Her complete blood count revealed a normal white blood cell (WBC) and platelet count, but low hemoglobin (11.5 g/dL) and hematocrit (34.0%). Her urine analysis was significant for a 2 + (41−80) leukocyte esterase, WBC count 132 / High-Power Field (HPF). Urine cultures were positive for Klebsiella pneumoniae, an extended-spectrum beta-lactamase producer. Sensitive to only amikacin, meropenem and imipenem. She was started on a 14-day course of meropenem 800 mg IV every 8 h (60 mg/kg/day). Her serum creatinine during hospitalization dropped from 136 to 67 mcmol/L (1.54–0.76 mg/dL). Her blood urea nitrogen initially decreased from 10 mmol/L to 7 mmol/L (28 mg/dL to 19.6 mg/dL), then increased to 14.2 mmol/L (39.8 mg/dL); however, this fluctuation was not considered clinically significant. After a 14-day course of meropenem, a subsequent urine culture was negative, and the patient was discharged. Daily intravesical amikacin instillation (250 mg dissolved in 50 mL of 0.9% normal saline [5 mg/mL]) was initiated and instilled into the urinary bladder via intermittent catheterization by the caregiver, with 2 h dwell time. The regimen was administered daily for 2 weeks, then on alternate days for 10 weeks, followed by twice weekly for 12 weeks to prevent recurrent UTIs [18]. At her 2-week follow-up visit, she was additionally started on sulfamethoxazole/trimethoprim (Bactrim DS) 80 mg orally once daily at home (2 mg/kg/day of trimethoprim) as prophylaxis. Approximately 18 months follow-up after discharge, the patient remained asymptomatic with no recurrent UTIs (Table 1).

**Table 1:** Previous isolated urine culture with antimicrobial susceptibility results.

**Episode**	**Organism Isolated**	**Antibiotic**	**MIC (µg/mL)**	**Interpretation**
1st	Klebsiella pneumoniae ESBL	Ampicillin		R
		Ciprofloxacin		S
		Gentamicin		S
		Imipenem		S
		Meropenem		S
		Nitrofurantoin		I
		TMP-SMX		R
2nd		Ciprofloxacin		S
		Gentamicin		S
		Imipenem		S
		Meropenem		S
		Nitrofurantoin		S
		TMP-SMX		R
3rd		Ampicillin		R
		Cefuroxime		R
		Ceftazidime		R
		Ertapenem		S
		Ciprofloxacin		R
		Gentamicin		S
		Imipenem		S
		Meropenem		S
		Nitrofurantoin		R
		TMP-SMX		R

Abbreviations: ESBL: extended-spectrum beta-lactamase producer, MIC: Minimum Inhibitory Concentration, R: Resistant, S: Susceptible, I: Intermediate.

## Discussion

This case report demonstrates the successful use of intravesical amikacin intillation as an adjunctive prophylaxis option for recurrent urinary tract infections (rUTIs) in a pediatric patient with neurogenic bladder and chronic kidney disease. Children with neurogenic bladder face an increased risk of rUTI due to impaired bladder emptying, use of catheters and inadequate bladder defenses. Standard preventive measures in these cases involve clean intermittent catheterization [7], antispasmodic therapy, and systemic antibiotics as prophylaxis; however, these preventive measures can fail when multidrug-resistant organisms are implicated [8].

Common agents for treating UTIs in patients with renal insufficiency may include carbapenams, cephalosporins, doxycycline, fosfomycin, penicillins, and quinolones, such as norfloxacin [9], [10], [11], [12], [13]. In adult patients with end-stage renal failure, quinolones (eg, ciprofloxacin, levofloxacin) are typically first-line agents, with cefdinir or cefpodoxime typically second-line agents [14], [15]. For prophylaxis, trimethoprim/sulfamethoxazole or Nitrofurantoin is the preferred agent [16]. MDR organisms that are resistant to all these agents will leave the clinician with very few options. In these scenarios, and in patients with concomitant significant renal insufficiency, the intravesical instillation of solutions of antibiotics- most commonly gentamicin- has been reported to avoid systemic toxicity while allowing high local drug concentrations directly into the bladder with minimal systemic dose [17].

Previously published pediatric case reports describing intravesical amikacin (other aminoglycosides may also be used) have a variety of instillation doses, schedules and clinical outcome measures that describe its clinical success for both eradicating MDR pathogens and reducing recurrence. The protocol in our patient (250 mg in 50 mL saline, daily treatments then tapering to twice weekly) [18] is higher in absolute dose than some previously published pediatric protocols (sometimes as low as 15–80 mg/dose) [19] however, the differences reflect patient size, organism susceptibility and the objective of achieving sustained local exposure in a patient with prior severe, recurrent, MDR infections. Case reports importantly demonstrate both treatment benefit and prophylactic benefit, with intravesical treatment in conjunction with systemic therapy for acute infection and followed by intravesical prophylaxis alone [18], [19].

Our results align with previous case reports and short series supporting the use of intravesical aminoglycosides in adult and pediatric patients with refractory UTIs. Wood, et al., described the use of continuous bladder tobramycin irrigation in a critically ill female patient with Enterobacter cloacae infection [20], and bladder instillations of amikacin for emphysematous cystitis caused by Escherichia coli [21]. Both reports noted successful bacterial eradication with intravesical therapy with systemic antibiotics. To their credit, more recent observational studies have also demonstrated that course of intravesical aminoglycosides result in decreased UTI recurrence rates, decreased UTI symptoms, and improved quality of life for patients, with clinically significant systemic absorption rarely being a problem—typically when the bladder mucosa is intact, and doses are monitored.

## Conclusion

In conclusion, intravesical amikacin bladder instillation represent a promising targeted and renal-sparing approach for the management of rUTIs from MDR organisms in patients with compromised renal function. This case contributes to the literature supporting the thoughtful use of this approach and the need for standardized protocols and larger prospective studies to better characterize appropriate dosing, duration and monitoring in children and adults.

## CRediT authorship contribution statement

**Yara A. Aldajani:** Writing – review & editing, Visualization, Validation, Investigation. **Sarah Alsubaie:** Writing – review & editing, Visualization, Validation, Investigation. **Afnan Alsunaidi:** Writing – original draft, Visualization, Validation, Supervision, Resources, Project administration, Methodology, Formal analysis, Data curation, Conceptualization. **Abdulaziz A. Alsalem:** Writing – review & editing, Writing – original draft, Visualization, Validation, Supervision, Software, Investigation, Data curation, Conceptualization.

## Author contributions

All authors contributed intellectually to the study conceptualization, data acquisition and compilation, statistical analysis, data interpretation, and drafting of the manuscript. All authors have read and agreed to the published version of the manuscript.

## Ethics approval/Consent

This case report has been approved by the Institutional Review Board (IRB) at King Saud University Medical City, Riyadh, Saudi Arabia, project number E−25–10253. The procedure does not pose “more than minimal risk to the human participant” per the assessment by the IRB.

## Funding

This research did not receive any specific grant from funding agencies in the public, commercial, or not-for-profit sectors.

## Declaration of Competing Interest

The authors declare that they have no known competing financial interests or personal relationships that could have appeared to influence the work reported in this paper.
